# A novel SAHA-bendamustine hybrid induces apoptosis of leukemia cells

**DOI:** 10.18632/oncotarget.4041

**Published:** 2015-05-08

**Authors:** Jing Yu, Shaowei Qiu, Qiufu Ge, Ying Wang, Hui Wei, Dianwu Guo, Shuying Chen, Shuang Liu, Shouyun Li, Haiyan Xing, Qing Rao, Jianxiang Wang, Min Wang

**Affiliations:** ^1^ State Key Laboratory of Experimental Hematology, Institute of Hematology and Blood Diseases Hospital, Chinese Academy of Medical Sciences and Peking Union Medical College, Tianjin, China; ^2^ Hangzhou Minsheng Institute of Pharmaceutical Research, Hangzhou, China

**Keywords:** histone deacetylase inhibitor, alkylating agent, hybrid, leukemia, apoptosis

## Abstract

Hybrid anticancer drugs are of great therapeutic interests as they can potentially overcome the deficiencies of conventional chemotherapy drugs and improve the efficacy. Many studies have revealed that the combination of histone deacetylase inhibitors (HDACi) and alkylating agents have synergistic effects. We reported a novel hybrid NL-101, in which the side chain of bendamustine was replaced with the hydroxamic acid of HDACi vorinostat (SAHA). NL-101 exhibited efficient anti-proliferative activity on myeloid leukemia cells especially Kasumi-1 and NB4 cells, accompanied by S phase arrest and caspase-3 dependent apoptosis. Importantly, it presented both the properties of HDAC inhibition and DNA damaging, as assessed by the acetylation of histone H3 and DNA double-strand breaks marker γ-H2AX. NL-101 also down-regulated the expression of anti-apoptotic protein Bcl-xL which was involved in the mitochondrial death pathway. Meanwhile, NL-101 induced apoptosis and DNA damage in primary cells from acute myeloid leukemia (AML) patients. NL-101 treatment could significantly prolong the survival time of t(8;21) leukemia mice with enhanced efficacy than bendamustine. These data demonstrate that NL-101 could be a potent and selective agent for leukemia treatment.

## INTRODUCTION

Despite the introduction of novel targeted therapies in the treatment of cancer, chemotherapies remain as primary therapy in clinical treatment for both solid tumors and hematologic malignancies. However, almost all chemotherapeutic agents give rise to severe toxicities and other undesirable side effects. The research for agents which have both remarkable potency and lower side effects is of significance.

Histone deacetylase inhibitors (HDACi) represent a novel class of targeted drugs which alter acetylation of chromatin-associated histones [1], as well as a range of non-histone substrates, including gene transcription factors involved in regulation of cell proliferation, migration and death [2]. Dysregulation of HDACs through over-expression or aberrant recruitment by fusion proteins, is commonly observed in leukemia and lymphoma [3, 4]. Several clinical trials have demonstrated HDACi had preferential efficacy in hematological malignancies with a good safety profile [5-7]. Vorinostat (Suberoylanilide hydroxamic acid, SAHA), belonging to the hydroxamic acid class, has been used for treatment of cutaneous T cell lymphoma with the FDA approval [8].

The synergistic effects of HDACi in combination with conventional alkylating agents have been validated in preclinical studies [9-11]. According to the recent research, HDACi synergistically enhanced the anticancer activity of bendamustine in multiple myeloma cells [12]. Bendamustine is a bifunctional compound, possessing the activity of alkylating and purine analogue agents. Clinical trials using bendamustine in combination with first line agents have shown notable results for treatment of hematological malignancies, including chronic lymphoid leukemia (CLL) and non-Hodgkin lymphoma [13-17]. In theory, hyperacetylation of histone proteins induced by HDACi could alter chromatin to an open structure, increase the accessibility of DNA and consequently potentiate the anticancer activities of DNA damaging agents [18-20]. Moreover, HDACi upregulate the acetylation status of DNA repair proteins and degrade their cytoprotective function [21, 22]. The above suggest the rationality of integrating the HDACi with bendamustine within a molecule. In this study, we reported the agent NL-101 which comprises those two chemoactive groups, and possess the dual mechanism of HDAC inhibition and DNA damaging. The compound exhibited potent anti-proliferative and pro-apoptotic activity towards AML cells both *in vitro* and *in vivo*.

## RESULTS

### The structural characteristics of NL-101

Bendamustine comprises a mechlorethamine (nitrogen mustard) group, a benzimidazole ring and a butyric acid side chain. The nitrogen mustard group acts to cross-link DNA strands and the butyric acid side chain to increases the water solubility [23, 24]. SAHA exerts its inhibitory effect via binding to the zinc domain in HDAC enzymes [25]. The six-carbon-long aliphatic chain terminated with a hydroxamic acid is essential for its HDAC binding. We postulated that introduction of the hydroxamic acid to bendamustine would keep the positive attributes of both drugs. Thus the compound was synthesized by replacing butyric acid in bendamustine by hydroxamic acid chain. For the sake of the patent protection, the chemical structural formula was not shown here.

### NL-101 inhibits cell proliferation in human leukemia cell lines

**Figure 1:**
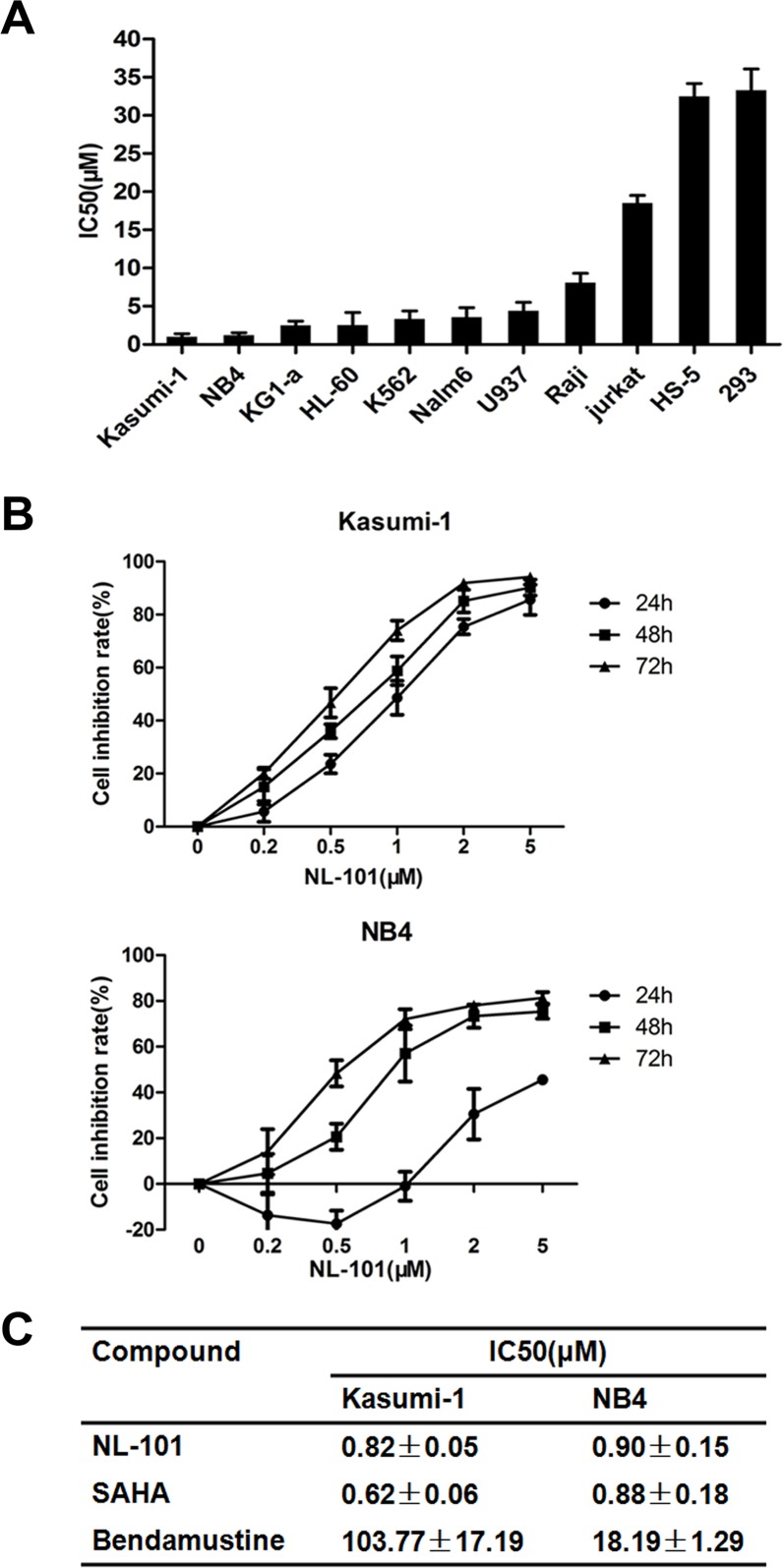
NL-101 inhibits cell proliferation of leukemia and lymphoma cell lines **A.** IC_50_ values obtained from MTT assay in leukemia and lymphoma cell lines treated with NL-101 for 48 h. HS-5 and HEK293 cells as normal non-malignant cell lines were also tested. Results are means±SD from at least three independent experiments. **B.** MTT assay of time- and dose-dependent effects on cell proliferation in Kasumi-1 and NB4 cells. The curves show the growth inhibition rate of NB4 and Kasumi-1 cells incubated with NL-101 at various concentrations (0.2∼5 μM) for indicated hours. **C.** IC_50_ concentrations of NL-101, SAHA and bendamustine in Kasumi-1 and NB4 cells for 48 h.

We first tested its biological effect on a broad range of human leukemia cell lines using MTT assay. Cells were exposed to NL-101 at concentrations ranging from 0.2 to 20 μmol/L, and fifty percent of inhibitiory concentration (IC_50_) was measured at 48 hours. As shown in Figure 1A, NL-101 exhibited anti-proliferation potency in all the leukemia cell lines examined. Kasumi-1 and NB4 cells were most sensitive and cell growth was inhibited in a dose- and time-dependent manner with the IC_50_ values of 0.82 μM and 0.9 μM at 48 h, respectively (Figure 1B). The growth of myeloid leukemia cells KG1-a, HL60 and K562 cells were also inhibited, and the sensitivity of malignant lymphoid cell lines Raji and Jurkat cells was relatively lower. Importantly, NL-101 treatment was tolerated by non-malignant cells HEK293 and HS-5 with the IC_50_ values more than 30 μM.

In addition, we compared the anti-proliferative effect of NL-101 with bendamustine and SAHA in Kasumi-1 and NB4 cells. The potency of NL-101 showed far superior to that of bendamustine, while similar with SAHA (Figure 1C). These results indicated that introducing HDAC inhibitory moiety may improve the efficacy of bendamustine. We further tested the combination of SAHA and bendamustine in myeloid leukemia cells and did find the synergism on the cell growth inhibition (Supplementary Figure S1).

### NL-101 induces S-phase arrest and caspase-3 dependent apoptosis in AML cells

**Figure 2:**
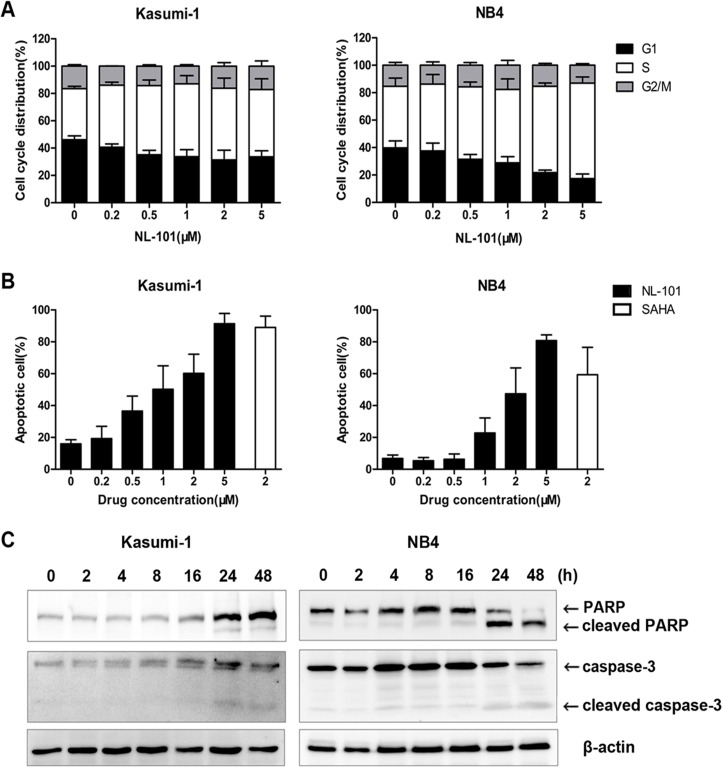
NL-101 induces S-phase arrest and caspase-3 dependent apoptosis in Kasumi-1 and NB4 cells **A.** Cell cycle analysis of Kasumi-1 and NB4 cells treated with different concentrations of NL-101 for 12 h. The percentage of cell cycle distribution was performed by flow cytometry and calculated by the ModFit program. **B.** Apoptosis analysis of Kasumi-1 and NB4 cells treated with NL-101 and SAHA for 48 hours. Apoptotic cells were determined by the flow cytometry using Annexin V/PI staining. Results are shown as mean percentage of annexin V^+^ cells from three independent experiments. **C.** Western blot analysis of PARP and caspase-3 in Kasumi-1 and NB4 cells exposed to 2 μmol/L of NL-101 for different time points. Arrowheads are cleaved PARP and caspases-3. β-actin was measured as a loading control.

Cell cycle analysis revealed that NL-101 induced S-phase arrest in Kasumi-1 and NB4 cells. As demonstrated in Figure 2A, the cell fraction at S-phase increased from (37.4±1.7)% to (52.5±7.4)% in Kasumi-1 cells and (44.9±6.1)% to (62.9±2.4)% in NB4 cells respectively, after exposure to 2 μmol/L of NL-101 for 12 hours. Moreover, an increase in the sub-G0/G1 cell population, which is the characteristic of apoptotic cells, was observed after more than 24 hours treatment especially in Kasumi-1 cells.

Apoptosis analysis further confirmed that NL-101 induced massive apoptosis in Kasumi-1 cells with majority of cells dead after 48 hours exposure; while in NB4 cells, treatment with 2 μmol/L of NL-101 killed nearly half of cells with levels slightly lower than that of SAHA (Figure 2B). Two apoptosis markers, cleaved caspase-3 and its substrate PARP were also detected in both cell lines after 24 h of treatment (Figure 2C).

### NL-101 induces HDAC inhibition and DNA damage

**Figure 3:**
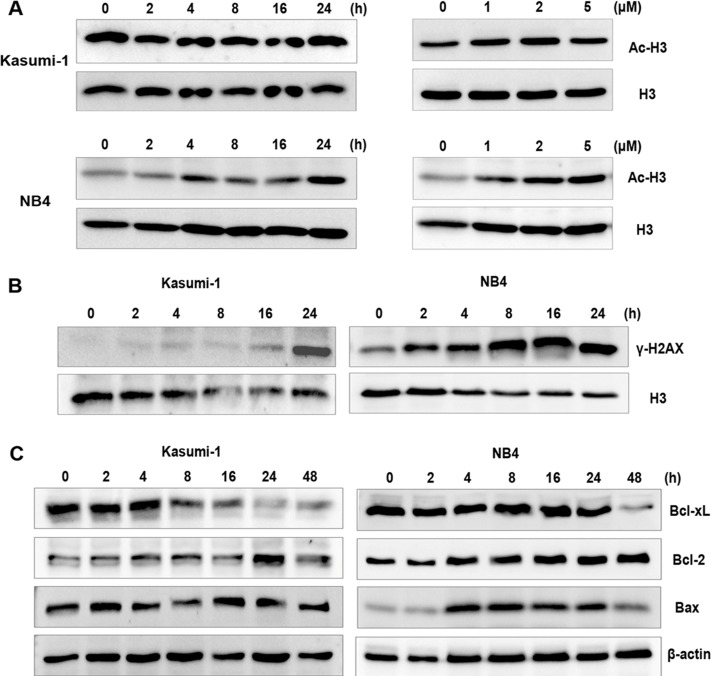
NL-101 presents dual-effects on histone acetylation and DNA damage **A.** Western blot analysis to detect the effect of NL-101 on acetylation of histone H3(Ac-H3). Cells exposed to 2 μmol/L of NL-101 for indicated hours (left panel) or incubated with variable concentrations for 48 h (right panel) were harvested, total histone H3 was determined as a loading control. **B.** Western blot analysis of the expression of γ-H2AX up to 24 h incubation with 2 μmol/L of NL-101. **C.** Western blot analysis of cell lysates from Kasumi-1 and NB4 cells using antibodies specific against Bcl-2, Bcl-xL and Bax.

To address whether the cytotoxic activities could be explained by the potency of two pharmacophores involved, we tested a possible HDAC inhibition and DNA damage effect of NL-101 by examining the level of acetylated histone protein and phosphorylation of H2AX (γ-H2AX) via Western blot analysis. As shown in Figure 3A, both cell lines with NL-101 treatment displayed moderate time- and dose-dependent increase in the acetylated histone H3. Meanwhile, NL-101 induced distinct accumulation of DNA damage marker γ-H2AX with prolongation of exposure time (Figure 3B).

Since the involvement of Bcl-2 family proteins in DNA damage-induced apoptosis has been well established, we monitored the expressions of the family members including anti-apoptotic proteins Bcl-xL, Bcl-2 and pro-apoptotic protein Bax. The expression level of Bcl-xL decreased in both cell lines and Bax increased in NB4 cells, while Bcl-2 displayed no change at the concentrations and time tested (Figure 3C). Besides, Bcl-xL decreased within hours of exposure and became obvious at 48 hours, prior to the caspase activation and apoptosis. These results suggest that the cytotoxity of NL-101 in AML cells could be attributed to the effect of HDAC inhibition and DNA damaging.

### NL-101 triggers apoptosis of leukemia cells from AML patients

**Figure 4:**
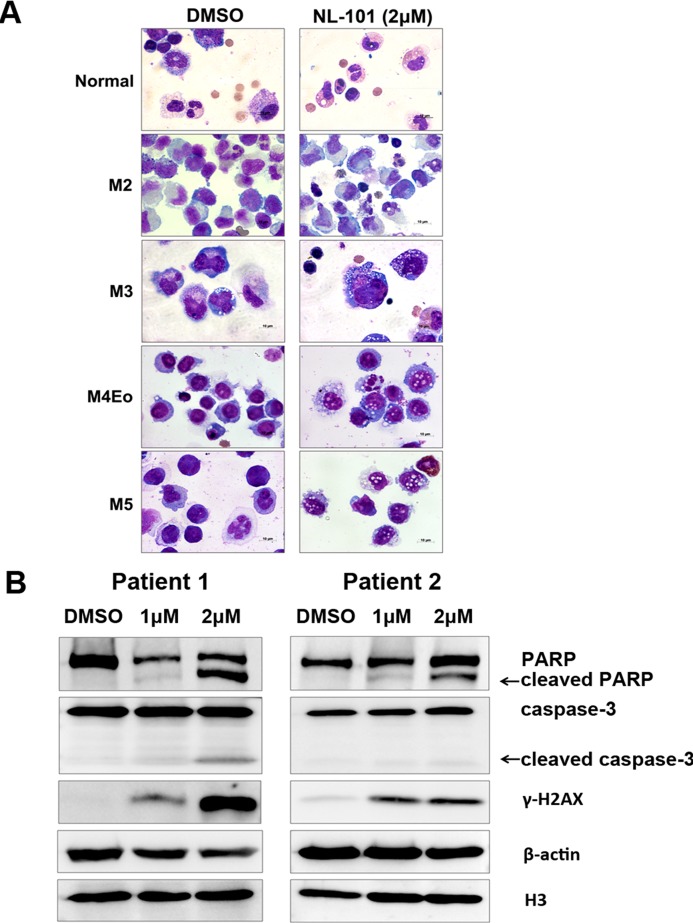
NL-101 triggers apoptosis of leukemia cells from AML patients **A.** Wright staining of cytosmears of blasts from bone marrow of AML patients and healthy donors after 48 h of treatment without/with 2 μmol/L of NL-101 (magnification, 100×). **B.** Western blot analysis of PARP, caspase-3 and γ-H2AX of treated BMMNCs. β-actin and H3 were measured as loading controls.

Given the pro-apoptotic properties exhibited in leukemia cell lines, we next determined the effects of NL-101 on human primary leukemia cells. Bone marrow mononuclear cells from 10 AML patients and three healthy donors were isolated and incubated with NL-101 for up to 48 hours. BMMNCs from patients showed morphologic characteristics of apoptosis, such as shrinking cytoplasm, nuclear fragmentation with intact cell membrane and vacuolar degeneration. In contrast, cells from normal adults were only slightly affected by NL-101 (Figure 4A). The apoptotic effects were also confirmed by the cleavage of PARP and caspase-3, and the increased γ-H2AX (Figure 4B).

### NL-101 prolongs survival of t(8;21) leukemia mice

**Figure 5:**
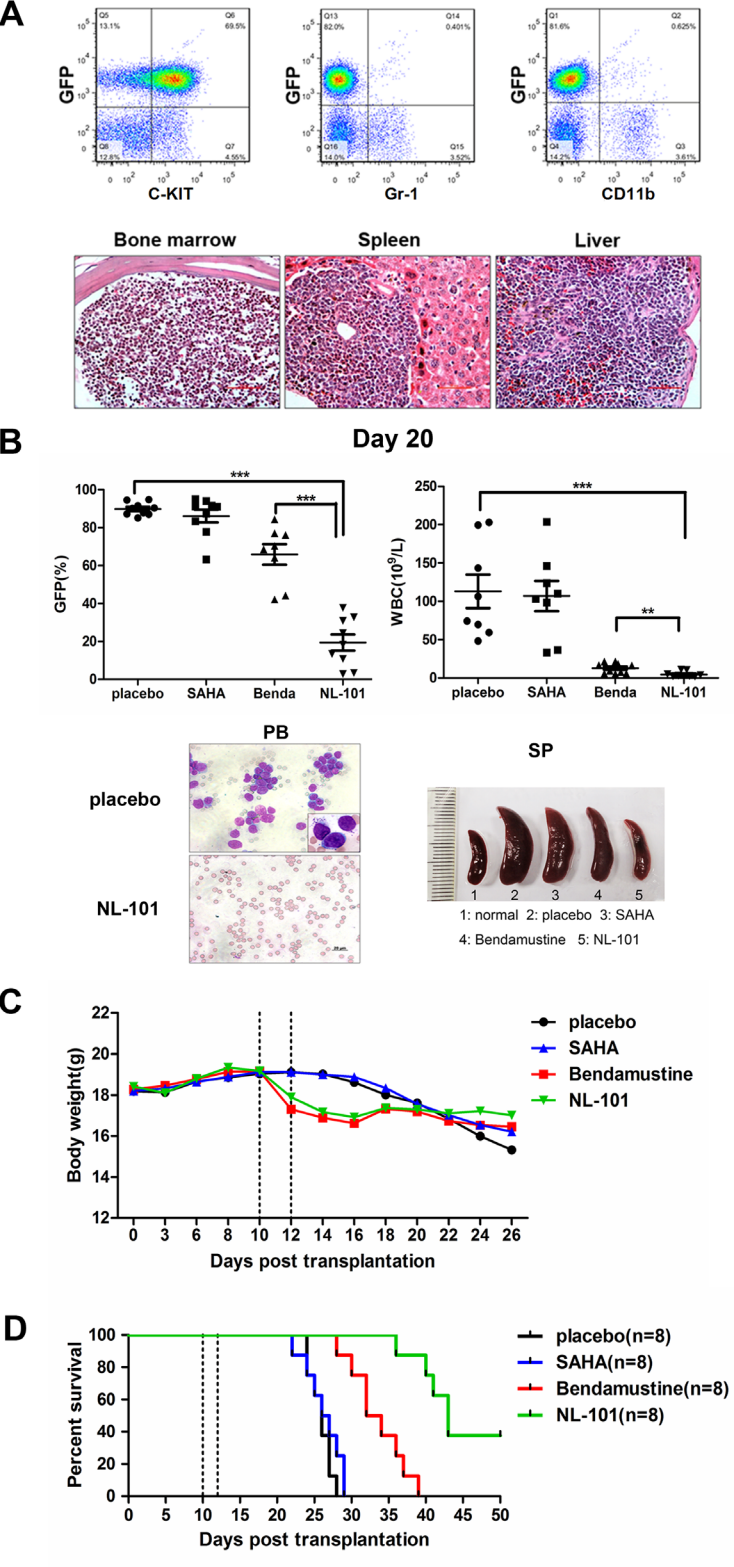
*In vivo* efficacy of NL-101 on murine leukemia models **A.** Immunophenotype analysis (upper panel) and histopathologic analysis (lower panel) of bone marrow from primary AE & C-KIT^D816V^ mice. **B.** Peripheral white blood cell (WBC) counts and flow cytometry of the circulating GFP^+^ cells in each group on day 20 (upper panel). Light microscopy of peripheral blood from mice treated with placebo and NL-101 (left in lower panel). The size of spleens from normal mice, t(8;21) leukemia mice treated with placebo, SAHA, bendamustine or NL-101, respectively (right in lower panel). The P-values were determined using Student's *t*-test, **P < 0.01, and ***P < 0.001. **C.**The average body weight of mice for different treatment groups during the observation period (n = 9). The dotted line represents the drug administration time. **D.** Kaplan-Meier survival curves of four groups. The *P*-values were determined by log-rank test, ***P < 0.001 versus control group.

To determine whether NL-101 was capable of suppressing leukemia development *in vivo*, we generated the transplantable mouse model coexpressing AML1-ETO and HyC-KIT^D816V^[26]. Recipient mice developed aggressive acute leukemia with extensive infiltrations of immature blast cells (C-KIT^+^, Gr1^−^, CD11b^−^) in hematopoietic organs (Figure 5A). For *in vivo* treatment, recipients of leukemia mice were administered NL-101 (30 mg/kg), bendamustine (30 mg/kg), SAHA (50 mg/kg) or placebo for 2 days beginning on day 10 after transplantation.

The mice body weight and GFP^+^ cells in peripheral blood were monitored during the observation period. On day 20, both the peripheral WBC count and the percentage of GFP^+^ cells decreased in NL-101 and bendamustine treated mice compared with that of placebo group. Splenomegaly was observed in mice treated with placebo, while the spleen size was nearly normal in NL-101 treated mice (Figure 5B). In addition, there was no significant difference in the average body weight between control and treated mice (Figure 5C). From approximately 3 weeks after transplantation, mice in the placebo and SAHA group succumbed to the disease rapidly, whereas mice treated with NL-101 and bendamustine showed a longer survival time. The median survival was 33 days among mice receiving bendamustine; while in the NL-101 group, there were 3 mice still alive at the end of the 50-day observation period (Figure 5D).

## DISCUSSION

There is evidence for the synergistic effect of combined DNA damaging agents and HDAC inhibitors on cancer cells. However, traditional combination therapy of two individual drugs may possess disadvantages, including pharmacokinetic differences and drug-drug interactions. Instead, the combination of two different acting compounds into one hybrid can convey the synergy effects and lead to an improved pharmacological potency. Here, we disclosed a novel compound NL-101 synthesized by combining the hydroxamic acid moiety and the structure of nitrogen mustard. In this context, HDACi activity is integrated into the skeleton of DNA alkylating agent. To systematically evaluate its activity, NL-101(NSC-751447) was tested in the NCI's anticancer drug screen against 60 human cancer cell lines [27]. The screen results demonstrated its cytotoxicity against a wide range of cancer cell lines, with IC_50_ similar to SAHA and 35-fold lower than bendamustine on average. In addition, COMPARE analysis showed that no compounds in the NCI's database had similar mechanisms as NL-101, indicating its unique features that compensate it from the conventional DNA alkylating agents and HDACi.

Our initial study *in vitro* demonstrated that NL-101 inhibited the growth of all the leukemia cells tested. Kasumi-1 and NB4 cells were most sensitive, and normal cell lines were more resistant to NL-101 treatment. Previous studies have demonstrated that HDAC inhibitors are potent particularly in AML with AML1/ETO and PML-RARα fusion proteins since they can recruit HDAC-containing complexes and provide targets for HDAC inhibitors [28]. In addition, HDACi are known for their selective cytotoxicity that discriminated between normal and malignant cells. These observations raised the question of whether NL-101 had the property of HDAC inhibition. We therefore examined the level of histone acetylation and found the accumulation of acetylated H3 in a concentration and time-dependent manner with the NL-101 treatment. These may suggest that NL-101 targeted the HDAC, and the effect of HDAC inhibition contributed to its anti-proliferative activities.

For the other aspect, bendamustine is known to induce DNA cross-links that initiates a DNA damage response [29, 30]. Our results showed that NL-101 could lead to the phosphorylation of H2AX, which is the early response after formation of DNA double-strand breaks (DSB) and important for recruiting DNA repair proteins [31-33]. In addition, NL-101 treatment resulted in S phase arrest and the degradation of anti-apoptotic protein Bcl-xL. S phase arrest is the prominent effect of DNA damage, providing time for the repair or cellular apoptosis [18]. Bcl-2 family proteins affect the mitochondrial death pathway and regulate the apoptotic process induced by DNA damage, responsible for the anticancer effect of both HDAC inhibitor and DNA damaging agent [34-36]. These findings demonstrate that NL-101 possesses the activity of both SAHA and bendamustine.

Finally, the potency of NL-101 on Kasumi-1 cells was further confirmed *in vivo*. For the murine experiment, AML1-ETO expression may not be sufficient for AML, but it could cooperate with activating mutations of C-KIT in the induction of fatal AML. Thus we used the mouse model coexpressing AML1-ETO and C-KIT^D816V^ to compare efficacy of NL-101 with bendamustine and SAHA. Our results indicate that NL-101 significantly inhibited the growth of leukemia cells and prolonged survival compared to the control. NL-101 also exhibited enhanced anticancer potency than bendamustine, which might represent that the introducing of HDACi moiety did enhance the effect of NL-101. SAHA application as single agent showed no efficacy in this aggressive leukemia model, and a better effect may be observed by increasing the dosage and the administration times. In addition, NL-101 was well tolerated in leukemia mice without significant weight loss.

In conclusion, the novel compound NL-101 inhibited the proliferation and induced cellular apoptosis of a wide range of leukemia cell lines. It also showed therapeutic response in blasts from AML patients and in t(8;21) murine model. Through the rational combination of HDAC inhibitors and alkylating agents, the potency of NL-101 appeared to exceed both bendamustine and SAHA. These results provide evidence that NL-101 would be effective and promising for the treatment of AML.

## MATERIALS AND METHODS

### Reagents

NL-101, bendamustine and SAHA were supplied by Hangzhou Minsheng Institute of Pharmaceutical Research (Hangzhou, China). Compounds were dissolved in dimethyl sulfoxide (DMSO, Sigma, USA) and diluted with cell culture medium for *in vitro* studies. DMSO was used as the vehicle control with a final concentration below 0.1% of total volume. For administration to mice, NL-101 and bendamustine was dissolved with normal saline to 3 mg/mL. SAHA was dissolved in a vehicle of DMSO and diluted freshly with PBS when used (final concentration of DMSO was 2%).

### Cell lines, primary AML samples and culture conditions

The following human cell lines were used: AML with t(8;21) translocation (Kasumi-1), acute promyelocytic leukemia (NB4 and HL-60), acute myelomonocytic leukemia (U937), acute myeloid leukemia (KG1-a), chronic myelogenous leukemia (K562), Burkitt's lymphoma (Raji), B-cell acute lymphoblastic leukemia (Nalm6), T-cell acute lymphoblastic leukemia (Jurkat), human embryonic kidney 293 cells and HS-5 stromal cells. Bone marrow samples of AML patients and healthy donors were obtained from the Institute of Hematology and Blood Diseases Hospital, Chinese Academy of Medical Sciences and Peking Union Medical College. An informed consent from each patient was provided. The characteristics of these patients are listed in Supplementary Table S1. Bone marrow mononuclear cells (BMMNCs) were isolated by density gradient centrifugation using Ficoll solution (TBD Science, China). Both leukemia cell lines and BMMNCs were cultured in RPMI 1640 supplemented with 10% fetal bovine serum.

### Growth inhibition assay and evaluation of drug synergy

Cellular growth inhibition was evaluated using the MTT assay. Cells were cultured in 96-well plates at a density of 2×10^3^ to 2×10^4^ cells and treated with various concentrations of drugs for 24, 48 and 72 hours. The control cells were exposed to DMSO at a concentration equal to that in drug-treated cells. MTT was added to a final concentration of 1 mM. After 4 hours, formazan crystals were dissolved by addition of 100 μl of 10% SDS and overnight incubation. Absorbance was measured with Synergy H4 Hybrid Microplate Reader (Biotek, USA) at 546 nm. The effect of combining SAHA with bendamustine on cellular growth inhibition was evaluated by calculating the combination index (CI) as described by Chou and Talalay [37]. CI values less than 1 represent synergistic effects.

### Flow cytometry

The drug effects on cell cycle distribution and apoptosis in AML cell lines were analyzed by flow cytometry (LSRII, BD, USA). For cell cycle assay, cells were treated for 12 h with/without drug, fixed in 70% ethanol for 24 h at 4°C, and stained with 20 μg/ml propidium iodide (Sigma, USA) containing 10 μg/ml RNaseA for 15–30 min. Cell apoptosis rate was quantified by double staining with Annexin-V and propidium iodide (BioLegend, USA), considering Annexin V-positive populations as apoptosis cells. To monitor GFP positive cells, peripheral blood of mice was collected in PBS containing 0.2% EDTA, and incubated in red blood cells lysis buffer (150 mM NH_4_Cl, 10 mM KHCO_3_, 0.1 mM EDTA) for 10 min before analysis. APC-conjugated C-KIT, PE-conjugated Gr-1 and PerCP-conjugated CD11b (all from BioLegend, USA) antibodies were used for immunophenotype test. Results were analyzed with FlowJo software.

### Western blot analysis

Harvested cells were resuspended in a lysis buffer containing 50 mM Tris/HCl (pH 7.5), 150 mM NaCl, 1% Triton X-100, 1% sodium deoxycholate, 0.1% SDS and 0.1 mM PMSF. Proteins were analyzed by SDS-PAGE and Western blotting. Membranes were incubated with primary antibodies against pan-acetyl-H3 (Millipore, USA), PARP [poly(ADP-ribose) polymerase], γ-H2AX (Ser139), caspases-3, Bcl-xL, Bcl-2, Bax, H3, β-actin (all from Cell Signaling Technology, USA). Blots were visualized using the ECL (Millipore, USA) with Image Quant LAS-4010 (GE Healthcare, USA).

### Murine model of t(8;21) leukemia and *in vivo* drug treatment

To establish the primary t(8;21) leukemia mouse model, hematopoietic stem cells were isolated from C57BL/6 mice and transduced twice with equivalent titer of retrovirus encoding MSCV-AML1/ETO-IRES-GFP and MSCV-HyC-KIT^D816V^-IRES-GFP [38]. The recipient C57BL/6 mice were lethally irradiated at 900 cGy and intravenously transplanted with the transduced cells. The mice developed fetal leukemia and leukemic cells could be transplanted serially.

For *in vivo* drug treatment, 5×10^5^ GFP^+^ spleen cells from moribund mice in the third generation were injected intravenously to sublethally irradiated mice. Ten days after cell implantation, mice were randomized into treatment and control groups. The treatment group received 30 mg/kg NL-101 or bendamustine intravenously, or 50 mg/kg SAHA intraperitoneally for 2 days. The control group received the equivalent volume of placebo. The leukemia development was assessed by peripheral white blood cell counts using the hematology analyzer (Sysmex XT-2000i, Japan) and percentages of circulating GFP^+^ cells by flow cytometry. The mice were weighed every other day during the experimental period to assess toxicity of the treatments. The overall survival was measured from the date of transplantation until death. All animal experiments were approved by the Institutional Animal Care and Use Committee of Peking Union Medical College.

### Statistical analysis

SPSS software (version 16.0) was used to calculate IC_50_ values. The comparisons were performed by Student's t-test analysis using GraphPad Prism (version 5.0). The lifespan of mice was analyzed by Kaplan-Meier methods and a log-rank test. *P*-values < 0.05 were considered statistically significant. Briefly, *P < 0.05, **P < 0.01, and ***P < 0.001 in comparison.

## SUPPLEMENTARY FIGURE AND TABLE


