# Supplementary material for: A novel SAHA-bendamustine hybrid induces apoptosis of leukemia cells
Source: Oncotarget. 2015 May 8;6(24):20121–31. doi: 10.18632/oncotarget.4041 (PMC4652992; doi:10.18632/oncotarget.4041)
Supplement: Supplementary file 1 [file oncotarget-06-20121-s001.pdf]

A novel SAHA-bendamustine hybrid induces apoptosis of leukemia cells

Supplementary material

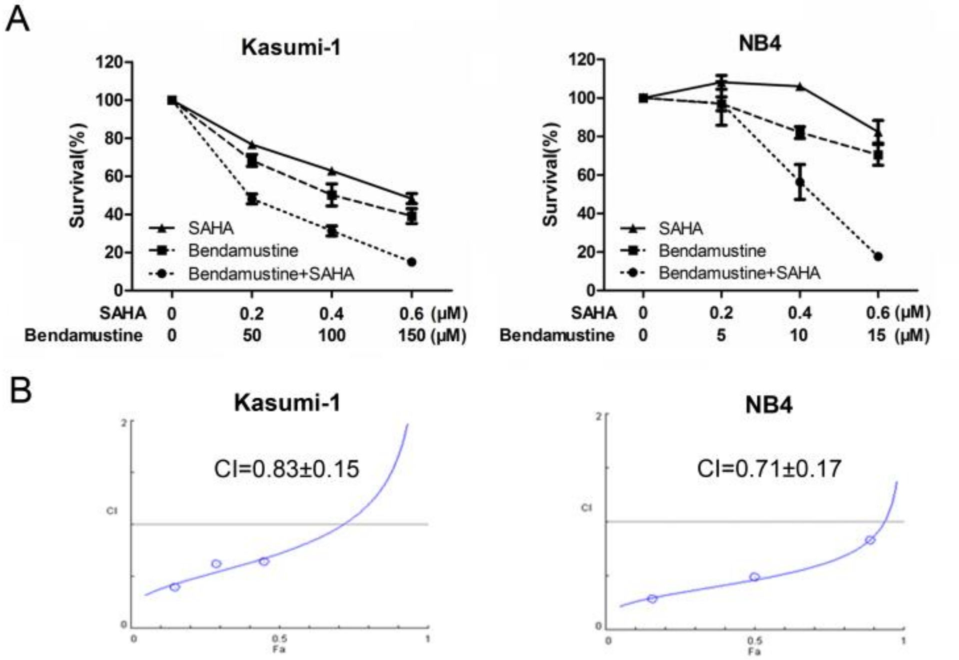

Supplementary Figure S1: SAHA and bendamustine cooperatively inhibit proliferation of leukemia cells. (A) MTT assay of bendamustine, SAHA and their combinations. Kasumi-1 and NB4 cells were treated with single drug or their combination in a fixed ratio for 48 h, the percentages of surviving cells were determined by MTT assay. (B) The combination index (CI) curves were calculated using CalcuSyn software. CI values less than 1 represent synergistic effects.

Supplementary Table S1: The information of the AML patients

Patients	Sex	Age	Subclassification (FAB)	Disease status
1	F	36	M2/t(8;21)	
2	M	46	M2/t(8;21)	
3	M	28	M3	
4	F	21	M3	newly diagnosed
5	M	22	M3	
6	M	47	M4Eo	
7	F	35	M5	
8	M	58	M2/t(8;21)	relapsed
9	M	38	M3	
10	F	34	M2/t(8;21)	remission
